# Moiré‐Assisted Realization of Octahedral MoTe_2_ Monolayer

**DOI:** 10.1002/advs.202304461

**Published:** 2023-10-22

**Authors:** Yasuaki Saruta, Katsuaki Sugawara, Hirofumi Oka, Tappei Kawakami, Takemi Kato, Kosuke Nakayama, Seigo Souma, Takashi Takahashi, Tomoteru Fukumura, Takafumi Sato

**Affiliations:** ^1^ Department of Physics Graduate School of Science Tohoku University Sendai 980‐8578 Japan; ^2^ Advanced Institute for Materials Research (WPI‐AIMR) Tohoku University Sendai 980‐8577 Japan; ^3^ Precursory Research for Embryonic Science and Technology (PRESTO) Japan Science and Technology Agency (JST) Tokyo 102‐0076 Japan; ^4^ Center for Science and Innovation in Spintronics (CSIS) Tohoku University Sendai 980‐8577 Japan; ^5^ Department of Chemistry Graduate School of Science Tohoku University Sendai 980‐8578 Japan; ^6^ International Center for Synchrotron Radiation Innovation Smart (SRIS) Tohoku University Sendai 980‐8577 Japan; ^7^ Mathematical Science Center for Co‐creative Society (MathCCS) Tohoku University Sendai 980‐8578 Japan

**Keywords:** angle‐resolved photoemission spectroscopy, moiré superlattice, molecular beam epitaxy, scanning tunneling microscopy, transition metal dichalcogenides

## Abstract

A current key challenge in 2D materials is the realization of emergent quantum phenomena in hetero structures via controlling the moiré potential created by the periodicity mismatch between adjacent layers, as highlighted by the discovery of superconductivity in twisted bilayer graphene. Generally, the lattice structure of the original host material remains unchanged even after the moiré superlattice is formed. However, much less attention is paid for the possibility that the moiré potential can also modify the original crystal structure itself. Here, it is demonstrated that octahedral MoTe_2_ which is unstable in bulk is stabilized in a commensurate MoTe_2_/graphene hetero‐bilayer due to the moiré potential created between the two layers. It is found that the reconstruction of electronic states via the moiré potential is responsible for this stabilization, as evidenced by the energy‐gap opening at the Fermi level observed by angle‐resolved photoemission and scanning tunneling spectroscopies. The present results provide a fresh approach to realize novel 2D quantum phases by utilizing the moiré potential.

## Graphical abstract

They propose a unique concept to realize originally unstable two‐dimensional materials by using moiré potential. This is demonstrated from their finding that octahedral MoTe_2_ which is unstable in the bulk form is stabilized on graphene due to the electronic‐energy gain associated with moiré superlattice. This concept would be useful to explore new crystal phases in various two‐dimensional materials.

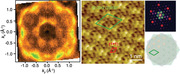

## 1 Introduction

Stimulated by the discovery of superconductivity^[^ ^1^ ^]^ and anomalous quantum Hall effect^[^ ^2^ ^]^ in twisted bilayer graphene, the search for exotic quantum phenomena by controlling the twist angle (magic angle) in bilayer systems is now becoming a central challenge in materials science. A key to realize such unique quantum states is the moiré superstructure potential which originates from the interference caused by phase mismatch between adjacent layers.^[^ ^3^ ^]^ Since the moiré structure has a much longer periodicity relative to that of the original lattice, the electronic band structure suffers from a strong modulation such as band folding and band hybridization,^[^ ^3^, ^4^, ^5^, ^6^, ^7^ ^]^ leading to exotic quantum phases such as superconducting, Mott‐insulating, and/or quantum spin‐liquid phases.^[^ ^1^, ^8^, ^9^, ^10^ ^]^ Besides graphene, transition‐metal dichalcogenides (TMDs) offer a useful platform to search for emergent physical properties in bilayers. This is because host TMDs themselves already have outstanding characteristics owing to many choices of constituting elements for transition metals and chalcogens, such as (i) stability in the 2D form due to van der Waals coupling between layers, (ii) rich electronic properties ranging from insulator to superconductor, and (iii) tunable spin‐orbit coupling and topological properties. Recently, the moiré superlattices of TMD bilayers were also reported to exhibit intriguing quantum properties, such as moiré‐trapped excitons in WSe_2_/WS_2_ and Mott‐insulator‐to‐superconductor transition in twisted bilayer WSe_2_.^[^ ^10^, ^11^ ^]^ In all of these engineering of moiré superlattices in graphene and TMDs, it is widely accepted that the crystal structure of host material remains unchanged even after the formation of moiré superlattice. Therefore, one can understand the electric structure of the moiré superlattice still based on that of the host materials. However, the following fundamental question arises; could the moiré potential alter the chemical bonding and modify the original crystal structure itself? If yes, this would create an otherwise unrealized novel crystal phase which may show various unprecedented properties.

**Figure 1:**
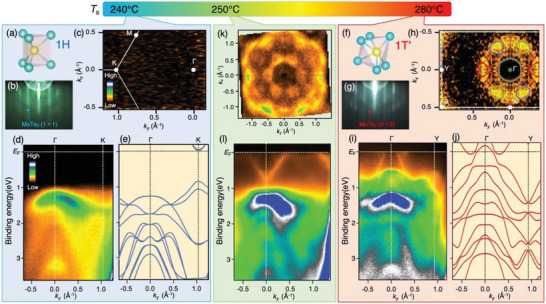
Selective fabrication of MoTe_2_ monolayers. a) Schematic crystal structure of monolayer 1H‐MoTe_2_. b) Reflection high‐energy electron diffraction (RHEED) pattern of monolayer 1H‐MoTe_2_ on BL‐graphene/6H‐SiC(0001) obtained at the substrate temperature *T*_s_ = 240 °C during the epitaxial growth. c) ARPES‐intensity mapping at *E*_F_ plotted as a function of 2D wave vector for monolayer 1H‐MoTe_2_. The intensity at *E*_F_ was obtained by integrating the ARPES intensity within ±20 meV of *E*_F_. d) Plot of ARPES intensity as a function of binding energy and *k_y_* (ΓK cut of graphene BZ) measured at *T* = 40 K. e) Calculated band structure along the ΓK cut for free‐standing monolayer 1H‐MoTe_2_. f–j), Same as (a‐e) but for monolayer 1T’‐MoTe_2_ obtained at *T*_s_ = 280 °C. k,l) FS mapping and ARPES intensity along the *k_y_* cut, respectively, for the film obtained at *T*_s_ = 250 °C.

Here, we propose a unique concept to realize new 2D materials by use of moiré potential. This concept is demonstrated by fabricating octahedral monolayer MoTe_2_ which has no counterpart in the bulk. We have chosen MoTe_2_ because it exhibits rich electronic phases depending on the type of crystal structures and the layer thickness,^[^ ^12^, ^13^, ^14^, ^15^, ^16^, ^17^, ^18^, ^19^, ^20^, ^21^, ^22^, ^23^, ^24^ ^]^ hetero‐bilayer systems containing MoTe_2_ are expected to have a high degree of freedom to manipulate the electronic states and physical properties. Bulk MoTe_2_ is known to be stabilized in two different types of crystal structures; the most stable 2H phase (**Figure** **1**a) with trigonal prismatic structure (D3h) and the quasi‐stable orthorhombic T_d_ phase (Pmn2) consisting of alternately stacked 1T’ layers rotated by 180° with each other. While bulk 2H‐MoTe_2_ is a semiconductor with an indirect band gap,^[^ ^14^, ^15^ ^]^ the monolayer counterpart (1H‐MoTe_2_) shows a direct band gap at the K point hosting the spin‐valley Hall effect.^[^ ^15^, ^16^ ^]^ On the other hand, bulk T_d_‐MoTe_2_ is a Weyl semimetal^[^ ^18^, ^19^ ^]^ whereas the monolayer (1T’‐MoTe_2_) is a candidate for a quantum spin Hall insulator (2D topological insulator).^[^ ^20^, ^21^, ^22^ ^]^ We fabricated a hetero‐bilayer system consisting of monolayer MoTe_2_ and bilayer graphene (BL‐graphene) on 6*H*‐SiC(0001) by MBE, and succeeded in realizing a novel structural phase different from so‐far discovered stable monolayer 1H‐ and 1T’ phases, namely the monolayer octahedral (1T) phase with a soft energy gap at the Fermi level (*E*_F_). Micro‐focused angle‐resolved photoemission spectroscopy (μ‐ARPES) and low‐temperature scanning tunneling microscopy (STM) in collaboration with first‐principles band‐structure calculations have revealed that this new 1T‐MoTe_2_ phase is realized by the band reconstruction due to the moiré potential formed between MoTe_2_ and BL‐graphene.

## 2 Results

We start by presenting the fabrication and characterization of monolayer MoTe_2_ films. Recently, insulating monolayer 1H‐ and metallic 1T’‐MoTe_2_ films were fabricated by a molecular‐beam‐epitaxy (MBE) method, and their electronic states were intensively investigated by spectroscopic techniques.^[^ ^22^, ^23^, ^24^, ^25^, ^26^ ^]^ In our case, by accurately controlling the substrate temperature *T*_s_ during the epitaxial growth, we have succeeded in selectively fabricating pure 1H and 1T’ phases of monolayer MoTe_2_, as summarized in Figure 1. When *T*_s_ was set to relatively low temperature of 240 °C, we observed the 1 × 1 streak pattern of reflection high‐energy electron diffraction (RHEED) image (Figure 1b; Figure S1, Supporting Information), indicative of the formation of 1H phase (Figure 1a). The plot of ARPES intensity at *E*_F_ as a function of in‐plane wave vector (Figure 1c) signifies no spectral weight at *E*_F_ over the entire momentum (*k*) space, showing the semiconducting nature of the fabricated film. In fact, the ARPES intensity along the ΓK cut of the 1H hexagonal Brillouin zone (BZ; Figure 1d) shows a negligible intensity within the binding energy (*E*_B_) range of 0–1 eV, demonstrating the existence of a band gap of ≈1 eV below *E*_F_. One can clearly see a hole band topped at the Γ point in the *E*_B_ range of 1–2 eV, together with a steeply dispersive hole band at 1.5–3.5 eV. These features are in overall agreement with the calculated band structure including the spin‐orbit coupling (SOC) for free‐standing monolayer 1H‐MoTe_2_ (Figure 1e; influence of the SOC is discussed in Section 2, Supporting Information), indicating the 1H‐phase nature of the film obtained at *T*_s_ = 240 °C (note that we observe the topmost valence band at the K point predicted in the calculation, although the intensity is relatively weak; see Figure S3, Supporting Information).

When the substrate temperature was elevated to *T*_s_ = 280 °C, the crystal and electronic structures show totally different characteristics. Instead of the 1 × 1 pattern, the RHEED image shows the 2 × 2 pattern (Figure 1g and Figure S1, Supporting Information) indicative of formation of 1T’ phase. This phase is regarded as a distorted 1T phase with the 2 × 1 periodicity, characterized by two inequivalent Te atoms with different Mo‐Te bond lengths (Figure 1f).^[^ ^13^ ^]^ In fact, the Te 4*d* core level spectrum shows an additional energy splitting due to the existence of two different types of bonding,^[^ ^27^ ^]^ unlike the case of 1T phase with no splitting (see Figure S4, Supporting Information). Due to the symmetry difference between 1T’‐MoTe_2_ (C_2_) and graphene substrate (C_6_), a 2 × 2 pattern appears in the RHEED image because three equivalent 1T’ domains rotated by 120° from each other coexist in the film. One can immediately recognize in Figure 1i that the ARPES intensity of this sample measured along the ΓY cut of the 1T’ rectangular BZ (same as the ΓK cut of the 1H hexagonal BZ) is more complicated than that of the 1H counterpart (Figure 1d). For example, we observe a hole band topped at ≈0.5 V, a rapidly dispersive hole band crossing *E*_F_ at slightly away from the Γ point, and a shallow electron band near this band (white arrow). These features are well reproduced in the band calculation with SOC for free‐standing 1T’‐MoTe_2_ (Figure 1j; see Section 2, Supporting Information for the influence of SOC). The Fermi‐surface (FS) mapping in Figure 1h is found to be consistent with the calculation for the 1T’ structure when taking into account the contribution from three domains rotated by 120° from each other. The assignment of 1H and 1T’ phases is also confirmed by the STM image, as discussed in Section 5 (Supporting Information). Thus, the present study establishes a method to selectively fabricate the 1H and 1T’ phases of monolayer MoTe_2_ by controlling accurately the substrate temperature (*T*_s_) during the epitaxial growth.

It is naturally expected that when *T*_s_ is set between 240 and 280 °C, the grown film would be composed of a mixture of 1H and 1T’ phases, as in the case of other monolayer TMDs such as 1H/1T‐NbSe_2_ and 1H/1T‐TaSe_2_.^[^ ^28^, ^29^, ^30^ ^]^ This is also expected from the growth condition of bulk MoTe_2_ crystals with the 2H and 1T’ structures.^[^ ^12^, ^13^ ^]^ Surprisingly, however, this prediction turned out to be not the case for monolayer MoTe_2_. As shown in Figure 1k, a complicated ARPES‐intensity pattern at *E*_F_, which is totally different from those of both 1H (Figure 1c) and 1T’ (Figure 1h) phases, shows up for the film fabricated at *T*_s_ = 250 °C. For example, the bright intensity region remarkably expands compared to the 1T’‐phase and a petal‐like structure newly appears around the Γ point. The difference is also clearly seen in the band structure; the film prepared at *T*_s_ = 250 °C exhibits a V‐shaped electron band centered at the Γ point (Figure 1l) while the 1T’ phase (Figure 1i) shows a Λ‐shaped hole band in the same momentum region. These experimental results suggest that the film fabricated at *T*_s_ = 250 °C does not belong to either the 1T’ phase or the 1H phase.

**Figure 2:**
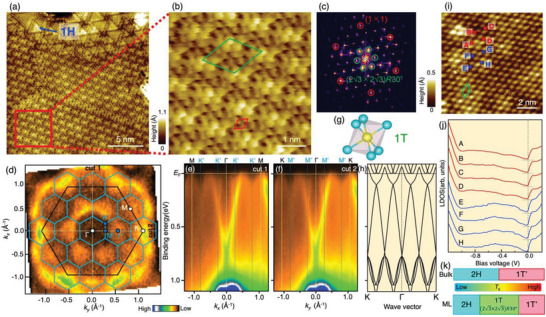
Electronic states of moiré superlattice consisting of monolayer 1T‐MoTe_2_ and BL‐graphene. a) Constant‐current STM image for a monolayer MoTe_2_ film obtained at *T*_s_ = 250 °C, in a surface area of 20 × 20 nm^2^ (sample bias voltage *V*_s_ = 1.5 V and set‐point tunneling current *I*_t_ = 100 pA). b) Expanded STM image (5 × 5 nm^2^) in the region indicated by a red square in (a). c) Fourier‐transform image of (b). Red circles show spots corresponding to the 1 × 1 unit cell of MoTe_2_, while green circles represent the (2√3 × 2√3)*R*30° superspot. d) Plot of ARPES intensity at *E*_F_ as a function of 2D wave vector. Solid black and blue hexagons indicate the BZs of the original 1 × 1 lattice and the (2√3 × 2√3)*R*30° superlattice, respectively. e,f) ARPES‐intensity plots as a function of *k_x_* (or *k_y_*) and *E*_B_ measured along cuts 1 and 2 shown by white dotted lines in (d). g) Schematic crystal structure of monolayer 1T‐MoTe_2_. h) Calculated band structure of 1T‐MoTe_2_ that takes into account band folding with the periodicity of (2√3 × 2√3)*R*30°. i) STM image in the surface area of 10 × 10 nm^2^ (sample bias voltage *V*_s_ = ‐1.0 V and set‐point tunneling current *I*_t_ = 500 pA). Green rhombus in (b) and (i) indicates the unit‐cell of the moiré superlattice. Note that the difference in the appearance of STM image between (a) and (j) is due to the difference in the *V*_s_ value. j) *dI*/*dV* spectra obtained at several positions indicated by crosses A‐H in (i). k) Schematic diagram of bulk and monolayer MoTe_2_ crystal phases as a function of growth temperature.

To obtain further insights into the origin of anomalous ARPES results, we performed STM for the sample fabricated at *T*_s_ = 250 °C. **Figure** **2**a displays the STM image in a relatively wide spatial region of 20 × 20 nm^2^ measured at *T* = 4.8 K, which mainly reflects the spatial distribution of Te‐5*p* electrons at the topmost surface. One can recognize that the upper side of the STM image is characterized by tiled triangular islands. Since a similar feature was observed in monolayer 1H‐MoSe_2_,^[^ ^31^ ^]^ these islands are attributed to the 1H phase formed in a small area of film. On the other hand, the remaining area covering ≈80% of the total STM image does not show a triangular pattern but exhibits a characteristic modulation with a longer periodicity. This is better visualized in the magnified STM image (Figure 2b) of the area shown by red square in Figure 2a. Whereas individual Te atoms are well resolved to show the 1 × 1 unit cell drawn by tracing the center of neighboring Te atoms (red rhombus), the intensity is periodically modulated to form a superstructure with a larger unit cell, as shown by green rhombus drawn by connecting Te atoms with brightest intensity. The Fourier transform image (Figure 2c) signifies clear (2√3 × 2√3)*R*30° superspots (green circles) corresponding to the superstructure unit cell shown in Figure 2b. Also, by looking at several STM images obtained under different measurement conditions for sample location, bias voltage, and tunneling current, we found that islands with the (2√3 × 2√3)*R*30° superstructure are almost homogenously distributed on a wide graphene area and its total area is ≈90% of the surface; for details, see Section 6 (Supporting Information).

Since the existence of (2√3 × 2√3)*R*30° superstructure is established by STM, we re‐examine the FS image and band structure observed by ARPES. As shown in Figure 2d, the FS image shows a good matching with the periodicity of (2√3 × 2√3)*R*30° superstructure; when the reconstructed BZ for the (2√3 × 2√3)*R*30° superstructure (blue hexagons) is overlaid on the ARPES intensity at *E*_F_, the observed petal‐like feature is well situated at the center of each reconstructed BZ. This suggests that the FS is largely modulated by the (2√3 × 2√3)*R*30° superstructure. As seen from the ARPES intensity along the ΓM/ΓK cut of the original 1 × 1 BZ (Figure 2e,f), the observed FS pockets are found to be formed by the highly dispersive V‐shaped bands with strong intensity and its replica‐like features with relatively weak intensity which are attributed to the folded bands associated with the (2√3 × 2√3)*R*30° superstructure.

## 3 Discussion

We have examined several possibilities to account for the emergence of unusual phase between the 1H and 1T’ phases, such as the edge state associated with the quantum spin Hall phase of monolayer 1T’‐MoTe_2_,^[^ ^20^, ^21^ ^]^ the mirror twin boundary state observed at the boundary of two different 1H domains in MoSe_2_,^[^ ^31^ ^]^ and other crystal phases such as Mo_5_Te_8_. However, all these possibilities are excluded (for details, see Section 7, Supporting Information). We concluded that the observed state originates from the 1T phase (Figure 2g) which is hardly obtained in the bulk due to its unstable nature. This is supported by the reasonable agreement of the ARPES results (Figure 2f) with the calculated band structure with SOC for the 1T phase (Figure 2h) that incorporates the band folding by the (2√3 × 2√3)*R*30° periodicity (we have confirmed that the folding of calculated bands in the 1H and 1T’ phases does not reproduce the ARPES results; the case for 1H phase is shown in Section 8, Supporting Information), as well as the absence of energy splitting in the Te 4*d* core‐level spectrum unlike the case of the 1T’ phase (see Figure S4, Supporting Information). We found that the reconstruction of BZ and resultant band folding associated with the (2√3 × 2√3)*R*30° superstructure led to opening of a soft energy gap at *E*_F_. This is seen from the *dI/dV* curves in Figure 2j obtained at several positions on the sample (points A‐H in Figure 2i). The V‐shaped density of states (DOS) in the bias voltage of ±0.1 eV is commonly observed in contrast to the high DOS at *E*_F_ in the calculation for the 1T phase (for details, see Section 9, Supporting Information). These results suggest that, despite the unstable nature of bulk 1T‐MoTe_2_, the 1T phase with the (2√3 × 2√3)*R*30° superstructure is stabilized in monolayer fabricated in a narrow growth temperature range between those of the 1H and 1T’ phases (Figure 2k). It is noted that the 1T‐phase nature needs to be further confirmed by crystal structure studies such as surface x‐ray diffraction (XRD) and scanning transmission electron microscopy (STEM). We leave such experiments as a challenge in future. It is also noted that the very narrow band gap and inherently low‐carrier semimetallic nature of the 1T phase may be potentially useful for application to electrical switching because an external perturbation would be able to efficiently change the band structure near *E*_F_. Obviously, more experiments such as alkali‐metal dosing and electrical gating to monolayer MoTe_2_ are required to examine this possibility.

Now that the basic electronic structure of monolayer MoTe_2_ is established, the next question is why the 1T phase is stabilized in the monolayer form unlike the case of bulk. A key to answer this question may reside in the mechanism to generate the (2√3 × 2√3)*R*30° superstructure. The (2√3 × 2√3)*R*30° superstructure may be driven by (i) the surface reconstruction associated with the dangling bond at the surface, (ii) the intercalation of Mo atoms, and (iii) the charge‐density wave associated with the FS nesting. Regarding (i), since no dangling bonds are expected to exist on the crystal surface of TMDs due to the van der Waals coupling between layers, the surface reconstruction associated with dangling bonds is unlikely. Regarding (ii), since we used exactly the same growth condition except for *T*_s_ to fabricate thin films, extra Mo atoms which could participate in the intercalation are hardly expected to exist only for the sample fabricated at intermediate *T*_s_. In addition, a van der Waals gap favorable for the intercalation does not exist in monolayer. Regarding (iii), the calculated FS for original 1T phase consists of small hole and electron pockets around the K point (see Figure S3, Supporting Information), but they do not satisfy the FS‐nesting condition with the (2√3 × 2√3)*R*30° superlattice vector.

**Figure 3:**
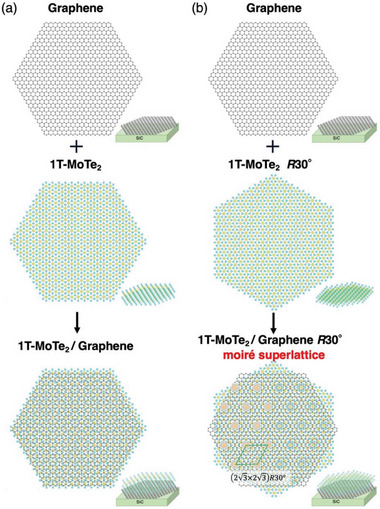
Configuration of crystal structure for monolayer 1T‐MoTe_2_ and graphene to realize moiré superstructure. a) Schematic crystal structure of (top) graphene, (middle) 1T‐MoTe_2_, and (bottom) their heterostructure with the rotation angle *θ* = 0°. b) Same as (a) but with *θ* = 30° that signifies the moiré pattern with the (2√3 × 2√3)*R*30° periodicity.

As a most plausible explanation for the origin of (2√3 × 2√3)*R*30° superstructure, we propose the moiré potential associated with the stacking of MoTe_2_ and BL‐graphene layers. A well‐known example of moiré potential is a homo‐bilayer of twisted bilayer graphene in which a small difference in the rotation angle *θ* (≈1°) between two adjacent graphene layers with the identical lattice constant produces a moiré structure and triggers the band folding to give rise to exotic physical properties such as superconductivity.^[^ ^1^ ^]^ In the present case, we fabricated a hetero‐bilayer with different host materials having different lattice constants (2.46 Å for graphene and ≈3.4 Å for MoTe_2_). Even in such an incommensurate case, a moiré pattern can be sometimes formed in a specific *θ*, as reported in other TMD/graphene hybrids.^[^ ^32^ ^]^ This is highlighted in **Figure** **3**, where the usual stacking of graphene and MoTe_2_ with *θ* = 0° does not produce a moiré pattern due to its incommensurate nature (Figure 3a), whereas the stacking with *θ* = 30° is nearly commensurate, exhibiting a clear moiré pattern with the (2√3 × 2√3)*R*30° periodicity, in striking agreement with the periodicity of our STM image. The growth of monolayer MoTe_2_ with rotation of crystal axis by 30° with respect to that of BL‐graphene substrate is confirmed by ARPES measurements (for details, see Section 10, Supporting Information). These arguments strongly suggest that the (2√3 × 2√3)*R*30° superstructure originates from the moiré structure.

It is generally known that TMD films can be fabricated on a BL‐graphene substrate even when the lattice matching is incommensurate, due to the van der Waals epitaxy nature.^[^ ^27^, ^28^, ^29^, ^30^, ^33^ ^]^ In such a case, the influence from the BL‐graphene substrate to the electronic states of TMD films is expected to be weak, as reported in many monolayer TMDs such as NbSe_2_ and TaSe_2_.^[^ ^28^, ^29^, ^30^ ^]^ On the other hand, in the present study, we utilize the commensurate lattice matching achieved by the rotation of MoTe_2_ layer by *θ* = 30° relative to BL‐graphene (It is noted that *θ* is fixed in the MBE growth and is not controllable, unlike the case of exfoliation). This enables us to grow the 1T phase which is otherwise difficult to be realized. Namely, the formation of moiré superstructure and the associated band folding lead to an energy gap opening at *E*_F_ due to the band hybridization (Figure 2j), and the resultant energy gain is responsible for the stabilization of 1T phase in monolayer. In this context, it is inferred that the 1H phase is unlikely to be stabilized by the moiré potential, because the total energy of monolayer 1H‐MoTe_2_ would not differ so much between *θ* = 0° and 30° due to the insulating nature (see Figure 1e). In this case, the band folding due to the moiré potential does not contribute to the additional electronic energy gain to stabilize the *θ* = 30° phase since the metallic bands are absent. This is consistent with the STM observation that locally signifies the *θ* = 30° 1H phase.^[^ ^24^ ^]^ Also, this is supported by the inconsistency between the APRES result and the calculated band structure that assumes the 1H phase including the band folding with the periodicity of (2√3 × 2√3)*R*30° (see Figure S8, Supporting Information). On the other hand, the *θ* = 0° 1T‐MoTe_2_ phase is unstable in a free‐standing form unlike the 1H phase, as can be inferred from the absence of 1T phase in the bulk crystal. However, the metallic band structure together with the energy gain associated with the band folding and the hybridization by the moiré potential effectively promote stabilization of the 1T phase with *θ* = 30°. Thus, although the *θ* = 30° lattices appear in both the 1H and 1T cases, their formation mechanisms are intrinsically different. Noticeably, the formation of 1T case is electronically driven and assisted by the moiré potential. Also, the 1T’ phase would not be stabilized by the moiré potential because 1T’‐MoTe_2_ has a different symmetry which does not favor the formation of moiré superstructure. The present study thus proposes a unique strategy to realize exotic crystal phases in epitaxial hetero‐bilayer systems by utilizing the moiré superstructure. This concept would be widely applicable to a variety of hetero‐bilayer systems to manipulate the electronic phase.

## 4 Conclusion

In conclusion, we have succeeded in selectively fabricating a stable monolayer 1H‐ and 1T’‐ MoTe_2_ on bilayer graphene by precisely tuning the growth substrate temperature in MBE. Moreover, we realized monolayer octahedral 1T phase with a soft energy gap at the *E*_F_ which is unstable in the bulk counterpart. By combining μ‐ARPES, STM, and first‐principles band‐structure calculation, we revealed that the electronic states of monolayer 1T‐MoTe_2_ is characterized by the band folding and Fermi‐surface reconstruction associated with the moiré potential of MoTe_2_/BL‐graphene commensurate lattice. The present finding opens a pathway to realize meta‐stable crystal phases by utilizing the moiré potential.

## 5 Experimental Section

A monolayer MoTe_2_ film was grown on a BL‐graphene substrate by the MBE method in an ultrahigh vacuum of 3 × 10^−10^ Torr. BL‐graphene was fabricated on the surface of an *n*‐type Si‐rich 6*H*‐SiC(0001) single crystal^[^ ^27^, ^28^, ^29^, ^30^, ^33^ ^]^ by resistive heating of the crystal at 1100 °C in a high vacuum better than 1 × 10^−9^ Torr. Then, monolayer MoTe_2_ was grown by evaporating molybdenum (Mo) atoms on the BL‐graphene/SiC substrate under tellurium (Te) atmosphere. The substrate was kept at 240–280 °C during the sample growth. The as‐grown film was annealed at ≈400 °C for 30 min. The growth process was monitored by the reflection high‐energy electron diffraction (RHEED). After the growth, the film was transferred to the ARPES measurement chamber without exposing it to air.

ARPES measurements were performed by using a DA‐30 electron energy analyzer with a micro‐focused synchrotron‐radiation at the beamline BL28A of Photon Factory (KEK). Circularly‐polarized light of *hν* = 100 eV with the beam‐spot of 10 × 12 µm^2[^ ^34^ ^]^ was used to excite photoelectrons. The energy and angular resolutions were set to be 25–50 meV and 0.2–0.3°, respectively. STM measurements were performed using a custom‐made ultrahigh vacuum (UHV) STM system^[^ ^35^ ^]^ at *T* = 4.8 K under UHV below 2.0 × 10^−10^ Torr. Te capping with ≈20 nm thick was deposited on MoTe_2_ film to protect the surface. This capping was removed in the STM chamber by Ar‐ion sputtering for 40 min and subsequent annealing at 250 °C for 120 min. All STM images were obtained in constant current mode with W tips.

First‐principles band‐structure calculations for monolayer MoTe_2_ were carried out by using Quantum‐Espresso code.^[^ ^36^, ^37^ ^]^ The generalized gradient approximation (GGA) ‐ with the Projector Augmented Wave (PAW) method realization using PSLibrary was used.^[^ ^38^ ^]^ The plane‐wave cutoff energy and charge cut‐off energy were set to be 60 Ry and 500 Ry, respectively. The uniform *k*‐point mesh was set to be 24 × 24 × 1. All the atoms are fully relaxed until the Hellmann‐Feynman force becomes lower than 1 × 10^−4^ eV Å⁻^1^. The atomic positions and lattice parameters are shown in Table S1 (Supporting Information). The spin‐orbit coupling (SOC) was also included, and all the calculated band structures were obtained with the calculation including the SOC. The thickness of inserted vacuum layer was set to be more than 15 Å to prevent interlayer interaction. The influence from the substrate such as the charge transfer and the bonding at the interface was also neglected, and the band dispersion for the free‐standing monolayers of 1H, 1T, and 1T’‐MoTe_2_ was simply calculated. Influence of the moiré potential presented in Figure 2h was included as a simple band folding without band hybridization (i.e., empty‐lattice approximation).

## Conflict of Interest

The authors declare no conflict of interest.

## Supporting information

Supporting Information

## Data Availability

The data that support the findings of this study are available from the corresponding author upon reasonable request.
