# Supplementary material for: Moiré‐Assisted Realization of Octahedral MoTe2 Monolayer
Source: Adv Sci (Weinh). 2023 Oct 22;10(34):2304461. doi: 10.1002/advs.202304461 (PMC10700228; doi:10.1002/advs.202304461)
Supplement: Supplementary file 1 — Supporting Information [file ADVS-10-2304461-s001.pdf]

ADVANCED SCIENCE
Open Access

Supporting Information

for *Adv. Sci.*, DOI 10.1002/advs.202304461

Moiré-Assisted Realization of Octahedral $MoTe_2$ Monolayer

Yasuaki Saruta, Katsuaki Sugawara, Hirofumi Oka, Tappei Kawakami, Takemi Kato, Kosuke Nakayama, Seigo Souma, Takashi Takahashi, Tomoteru Fukumura and Takafumi Sato**

Supporting Information

Moiré-assisted realization of octahedral $MoTe_2$ monolayer

*Yasuaki Saruta, Katsuaki Sugawara *, Hirofumi Oka, Tappei Kawakami, Takemi Kato, Kosuke Nakayama, Seigo Souma, Takashi Takahashi, Tomoteru Fukumura, and Takafumi Sato *.*

S1. Comparison of the RHEED patterns between monolayer 1H and 1T' $MoTe_2$.

Figures S1a and S1b show a direct comparison of the RHEED images between the 1H and 1T' phases obtained at T_s = 240 °C and 280 °C, respectively (same as Figures 1b and 1g in the main text). From the comparison of line profiles between the two phases in Figure S1c, we found that the T_s = 240 °C sample (blue curve) contains only 1×1 peaks consistent with the undistorted 1H phase, whereas the T_s = 280 °C sample (red curve) shows additional peaks between the main 1×1 peaks (red arrows). This is associated with the 2×2 superstructure originating from three 2×1 1T' crystal domains rotated by 120° from each other. Such a critical difference in the lattice periodicity is consistent with the STM image for the 1H and 1T' phases as detailed in Section 5 and Figure S5 where the triangular image for the 1H phase and the rectangular image for the 1T' phase can be well identified, consistent with the RHEED pattern and the previous literatures [1-3].

S2. Spin-orbit interaction in the DFT calculations.

All the calculated band structures were obtained by including the SOC unless specified. As shown in Figure 1e of the main text, the influence of the SOC for the 1H phase can be identified as a small doubling (spin splitting) of some bands associated with the space-inversion symmetry breaking and SOC in monolayer 1H crystal. Such a splitting disappears at the Γ point due to the Kramers degeneracy protected by the time-reversal symmetry. On the other hand, in the case of 1T and 1T' phases, the energy bands do not show spin splitting because the crystal preserves the inversion symmetry. In this case, it is useful to clarify the influence of SOC by directly comparing the calculated band structure with and without SOC. We show in Figure S2 calculated band structures for monolayer 1T-$MoTe_2$ (a) without and (b) with SOC. While the overall valence-band structure is similar between the two cases, the inclusion of SOC lifts the band degeneracy at the band-crossing point, leading to the opening of a small spin-orbit gap. This spin-orbit gap is clearly visible around the K point at the intersection of the valence-band

top and the conduction-band bottom around E_F. As a result of the spin-orbit gap, the valence-band top becomes flatter. It is noted that the band folding shown in Figure 2h of the main text was carried out based on the calculation including the SOC shown in Figure S2b.

S3. Valence-band structure of monolayer 1H-$MoTe_2$.

Figure S3 shows the ARPES intensity along the ΓK cut for monolayer 1H-$MoTe_2$ fabricated at the substrate temperature T_s = 240 °C. One can recognize a hole band topped at the Γ point in the binding energy (E_B) range of 1-2 eV, which shows an overall agreement with the band structure calculated for free-standing monolayer 1H-$MoTe_2$ (Figure S3b). We found that the ARPES intensity of valence-band top at the K point predicted in the calculation is weak in the experiment due to the matrix-element effect of photoelectron intensity. When we take the second derivative of ARPES intensity to enhance such weak features (Figure S3c), a hole-like band topped at the K point shows up around the E_B of 1.2 eV in good agreement with the calculation. These results are consistent with the previous report that 1H-$MoTe_2$ is a spin-valley locked semiconductor produced by the space-inversion-symmetry breaking [4].

S4. Comparison of Te 4*d* core-level spectra.

The 1T structure obtained at T_s = 250 °C is also corroborated by the Te 4*d* core-level photoemission spectrum. As shown in Figure S4, the Te 4*d* core-level spectrum for the sample fabricated at T_s = 250 °C consists of two peaks which correspond to the spin-orbit satellites of Te $4d_{3/2}$ and $4d_{5/2}$ orbitals, respectively. On the other hand, for the sample obtained at T_s = 280 °C, each spin-orbit satellite peak appears to further split into two subpeaks. This is due to the existence of two different chemical bond lengths surrounding the Te atom, as naturally inferred from the 1T' structure [5]. The absence of such energy splitting in the sample obtained at T_s = 250 °C suggests that there is only one type of bond length in it, as expected from the 1T structure.

S5. STM images in monolayer 1H, 1T, and 1T' phases of $MoTe_2$

Figures S5a-c show a side-by-side comparison of the STM images in monolayer $MoTe_2$ among 1H, 1T, and 1T' phases, obtained with the substrate temperatures of T_s = 240-280 °C. On can clearly recognize triangular and rectangular shaped STM images for the 1H, and 1T' phases, respectively, in accordance with their expected lattice symmetries, consistent with the previous reports [3,6,7]. On the other hand, the STM image of the 1T phase exhibits a

$(2\sqrt{3}\times2\sqrt{3})R30°$ periodicity associated with the moiré superlattice, obviously different from the images of the 1H and 1T' phases.

S6. STM images for the T_s = 250 °C sample at different measurement conditions.

To clarify the quality of the film obtained at T_s = 250 °C, we extensively surveyed the sample surface with different measurement conditions including sample location, bias voltage V_s, and tunneling current I_t. As a result, we found that the 1T phase is a dominant phase. Figures S6a and S6b show representative STM images obtained at the sample positions different from those presented in Figures 2a, 2b, and 2i of the main text (note that V_s and I_t values are also different; see caption to Figure S6). Similarly to the case of Figure 2a, one can see in Figure S6a the superstructures with a $(2\sqrt{3}\times2\sqrt{3})R30°$ periodicity (note that the moiré superlattice unit-cell is indicated by green rhombus), whereas the local distribution of the bright and dark intensity area within the superlattice unit-cell is different from that of Figure 2a; this is likely because the two images were obtained at different V_s and I_t values. To clarify the periodicity of obtained intensity modulation, we carried out Fourier transform of Figure S6a. The obtained result in Figure S6c shows the $(2\sqrt{3}\times2\sqrt{3})R30°$ superstructure spots besides the 1×1 spots, as in the case of Figure 2c which was obtained by the Fourier transform of Figure 2b. We have confirmed in the T_s = 250 °C sample that the $(2\sqrt{3}\times2\sqrt{3})R30°$ superstructure appears irrespective of the measurement conditions, as also highlighted by the observation of superstructure in Figures S6b and S6d measured with different sample positions, V_s, and I_t (note that remaining spots which are not marked by the open red or green circles are also attributed to the $(2\sqrt{3}\times2\sqrt{3})R30°$ spots). Thus, the common appearance of the $(2\sqrt{3}\times2\sqrt{3})R30°$ superstructure in both the STM and Fourier-transform images irrespective of the measurement conditions strongly supports its reproducible and intrinsic nature.

We found that the 1H phase sometimes appears in a narrow region of the scanned surface area, as can be seen from the upper region of Figure 2a and the lower right region of Figure S6b. However, we found that the 1T phase always dominates the surface area. This is consistent with our ARPES data showing a predominant intensity from the 1T phase around E_F, as clarified in Figures 2g and 2h. By looking at several obtained STM images, we found that the 1T phase is almost homogenously distributed on a wide graphene area and its total area is approximately 90% of the surface, supporting that the 1T phase is a primary product and is not a metastable phase.

S7. Origin of unusual electronic states in $MoTe_2$ monolayer fabricated at T_s = 250°C.

We discuss the origin of unusual electronic states characterized by the complicated intensity pattern in the Fermi-surface (FS) mapping (Figure 2d) and the (2√3×2√3)R30° superstructure in the STM image (Figure 2b). One possibility is the topological edge state associated with the quantum spin Hall insulator phase of monolayer 1T'-$MoTe_2$ [8]. In such a case, the spectral intensity from the edge state is expected to be much weaker than that of the main two-dimensional (2D) bulk valence band. Also, the edge state should show a purely one-dimensional (1D) energy dispersion in ARPES and a spatially localized nature in STM.

Since all of these expectations are incompatible with our observation of the 2D FS in ARPES and the delocalized (2√3×2√3)R30° pattern in STM, this possibility is ruled out. Second possibility is the mirror twin boundary state which was reported to show up at the boundary of two 1H crystal domains in monolayer 1H-$MoSe_2$, exhibiting an electron-like dispersion at the Γ point [9]. However, this possibility is also ruled out because the mirror twin boundary state is expected to show a purely 1D dispersion in contradiction to the observed 2D nature. Third possibility is the state associated with a crystal with different chemical composition, i.e. Mo_5Te_8 [10]. Mo_5Te_8 has a 2D crystal structure as in other TMDs, and is known to be formed by introducing many Te vacancies to 1H-$MoTe_2$ [10]. This possibility is also excluded because the atomic image of STM does not support the formation of Mo_5Te_8, and the ARPES-derived band dispersion (V-shaped electron band centered at the Γ point) is obviously different from the calculated band dispersion for Mo_5Te_8 (hole-like dispersion at Γ [10]).

As a last and most plausible possibility, we propose the state originating from the 1T phase. Figure S7 shows (a) the valence-band ARPES intensity compared with (b) corresponding calculated band structure for monolayer 1T-$MoTe_2$ (without taking into account the moiré potential) in the valence-band region. One can see a rough agreement in the overall band dispersion in the binding-energy (E_B) range of 1-3.5 eV, such as a few holelike bands topped at the Γ point. On the other hand, a prominent V-shaped electron band and a much weaker hole band at $E_B \sim E_F$ -1eV around the Γ point appear to show no counterpart in the calculation. When calculated energy bands are folded by following the (2√3×2√3)R30° periodicity of moiré potential associated with the commensurate lattice matching between $MoTe_2$ and graphene, one can find a reasonable matching between the experiment and calculation, as detailed in Figure 2h of the main text. This supports the 1T nature of the film fabricated at T_s = 250°C.

S8. Comparison of ARPES intensity for the T_s = 250 °C film with the calculated band structure of monolayer 1H-$MoTe_2$

Previous experiments reported that the STM image shows the $(2\sqrt{3}\times2\sqrt{3})R30°$ periodicity even for the 1H phase [3,6]. It is thus important to show that the new phase (i.e. 1T phase) obtained in this study is different from it. A simple way to examine this point is to compare the experimental band structure with the calculated band structure that assumes the 1H phase including the band folding with the $(2\sqrt{3}\times2\sqrt{3})R30°$ periodicity. As shown in Figure S8, the calculated band structure for the this moiré 1H phase (Figure S8b) shows a large band gap in the binding-energy (E_B) range of E_F-0.9 eV as in the case of the simple 1H phase (see Figure 1e of the main text), in sharp contrast to the experimental data showing highly dispersive bands in this E_B range (Figure S8a). Obviously, the experimental band structure is more consistent with the calculated band structure for the moiré 1T phase (Figure S8c). Thus, the present experiment excludes the possibility that the new crystal phase obtained at $T_s = 250$ °C is a moiré 1H phase previously reported in refs. 3 and 6.

S9. Energy gap opening associated with moiré potential.

In addition to the STM observation of an energy gap (Figure 2j), we also observed it by ARPES. Figure S9b shows the energy distribution curves (EDCs) at $T = 40$ K symmetrized with respect to the Fermi level (E_F), measured at several **k** points on the ΓM (k_x) line (cuts 1-5 in Figure S9a). A clear gap opening is seen at the cut 2. This behavior is in sharp contrast to the calculated DOS which shows a monotonic increase on approaching E_F in the occupied region (Figure S9c). We also found that the energy gap shows a strong *k* dependence; the gap is not so clear for cuts 1, 3, and 5 in contrast to cuts 2 and 4. This supports the *k*-dependent band hybridization expected from the band folding picture associated with the moiré potential.

S10. 30°-rotated growth of monolayer 1T-$MoTe_2$.

We carefully checked the experimental geometry of the graphene/SiC(0001) substrate before the MBE growth and the $MoTe_2$ sample after the growth. We have confirmed the growth of monolayer $MoTe_2$ with rotation of crystal axis by 30° with respect to that of graphene substrate, by the FS mapping with ARPES. Figure S10a shows a photograph of sample holder with the SiC(0001) substrate. We aligned the ΓM and ΓK directions of SiC (and also graphene) to the horizontal and vertical axes, respectively. We have checked this geometry with the rough FS mapping of bilayer graphene grown on SiC(0001), by specifying the position of π*-band-derived small FS at the K point, as shown in Figure S10b (corresponding crystal geometry of graphene in real space is also shown in Figure S10c). After the growth of monolayer 1T-$MoTe_2$ onto this substrate, we have confirmed that the ΓK direction of the original 1×1 Brillouin zone

(BZ) for $MoTe_2$ lies along the horizontal direction, as shown by the FS mapping and corresponding BZs in Figure S10d (same as Figure 1k in the main text). This result supports the rotation of monolayer 1T-$MoTe_2$ by 30° relative to graphene, as illustrated in Figures S9c and S10e.

S11. Atomic positions and lattice parameters in the DFT calculations for $MoTe_2$

Table S1 shows a list of atomic positions and lattice constants for the 1T phase adopted in our calculations together with those for the 1H and 1T' phase. These results would serve as a useful reference for the crystal-structure analysis that aims to experimentally verify the 1T phase.

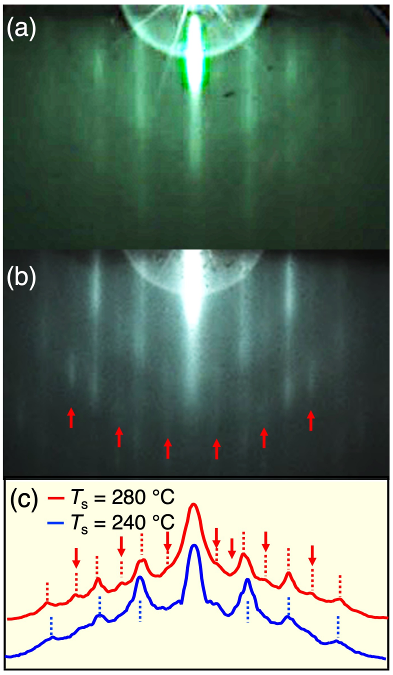

Figure S1. a, b) RHEED patterns of monolayer $MoTe_2$ films grown at T_s = 240 ºC and 280 ºC, respectively. (c) Direct comparison of RHEED intensity profiles between (a) and (b). Red arrows indicate superstructure streaks/peaks.

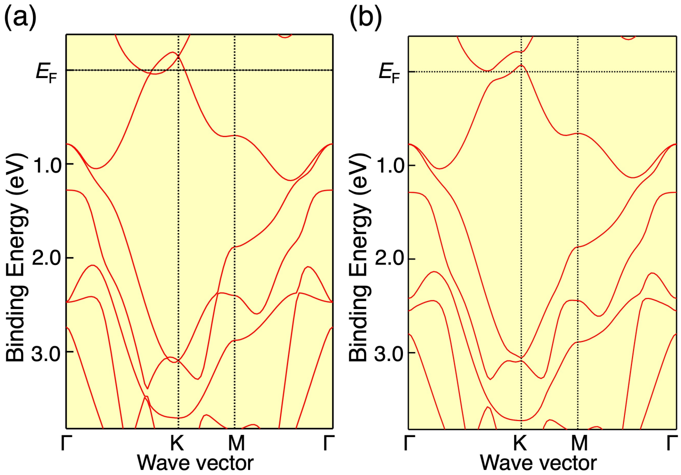

Figure S2. Calculated band structures for monolayer 1T-$MoTe_2$ (a) without and (b) with SOC along ΓKM high-symmetry line in hexagonal Brillouin zone.

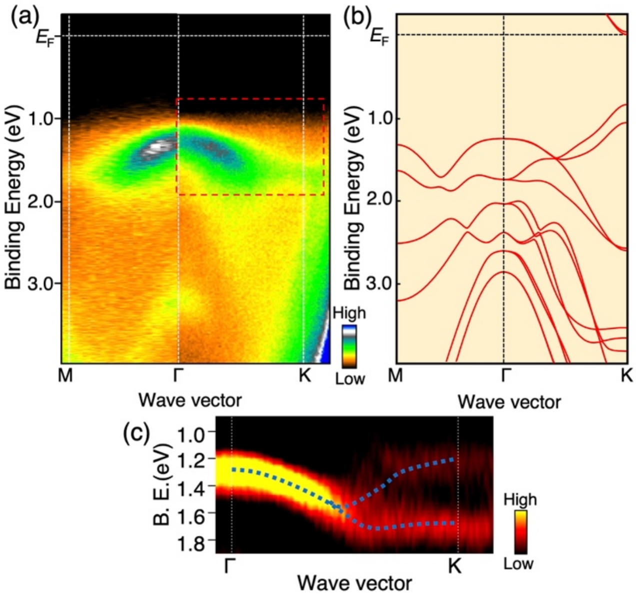

Figure S3. Band dispersion around the K point. a) Plot of ARPES intensity as a function of binding energy and k_y (MΓK cut) measured at T = 40 K. b) Calculated band structure along the MGK cut for free-standing monolayer 1H-$MoTe_2$. c) Plot of second-derivative ARPES intensity in the (E, k) region indicated by a dashed red rectangle in (a). Light blue curves are a guide for the eyes to trace the band dispersion.

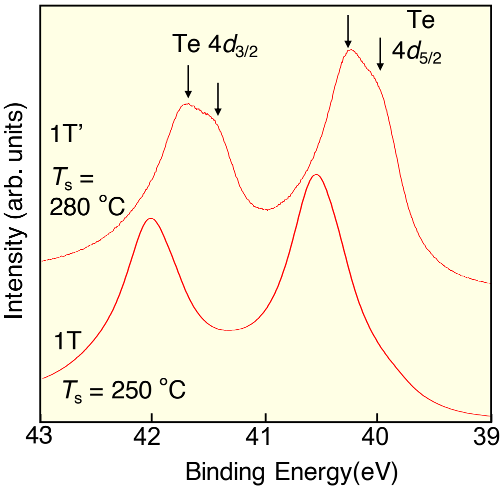

Figure S4. Comparison of Te 4d core-level photoemission spectrum measured with hv = 100 eV for monolayer $MoTe_2$ films obtained at T_s = 280 and 250 °C, corresponding to the 1T' and 1T phases, respectively.

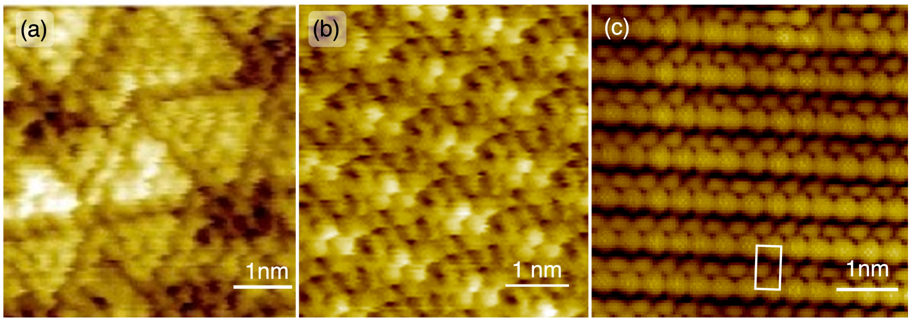

Figure S5. a-c) STM images of monolayer (a) 1H-, (b) 1T-, and (c) 1T'-$MoTe_2$ films.

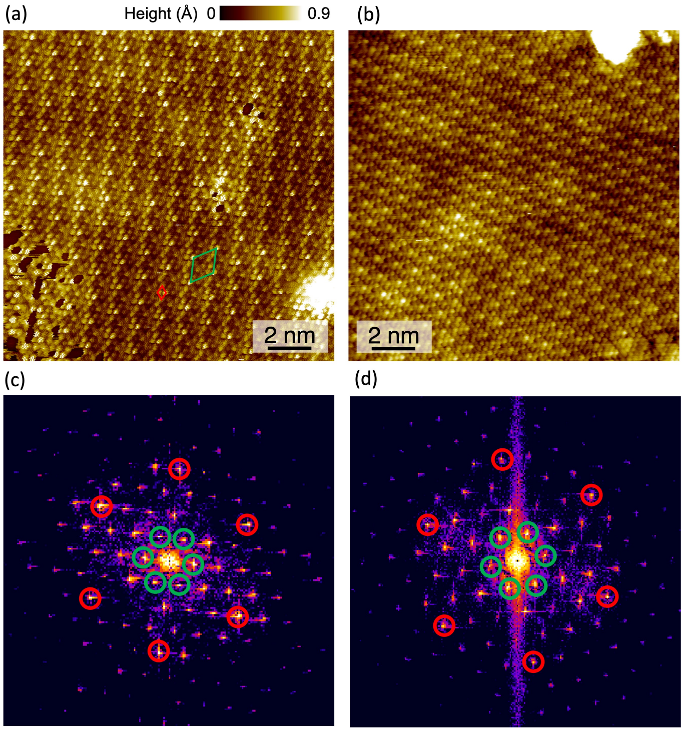

Figure S6. a, b) STM images obtained at the sample positions different from those presented in Fig. 2a. STM data were obtained with V_s = -0.1 V and I_t = 500 pA and (b) V_s = 1.5 V and I_t = 50 pA. c, d) Fourier-transform images of (a) and (b), respectively. Red and green circles correspond to the (1×1) and (2√3×2√3)R30° spots, respectively.

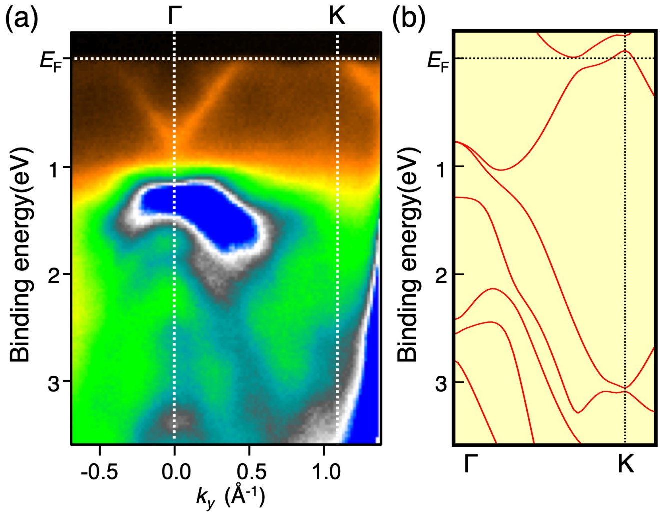

Figure S7. a) Plot of ARPES intensity as a function of E_B and k_y (ΓK cut of graphene BZ) measured at $T = 40$ K for monolayer 1T-$MoTe_2$. b) Calculated band structure along ΓK cut for free-standing monolayer 1T-$MoTe_2$.

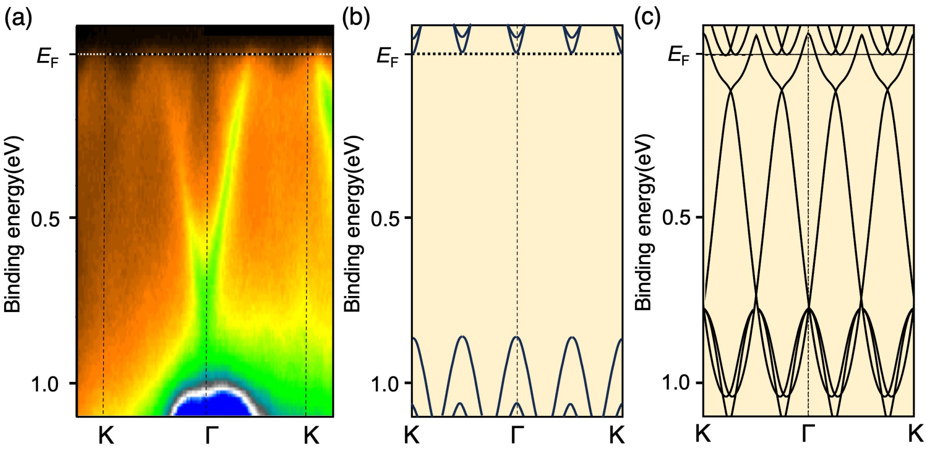

Figure S8. a) ARPES intensity of monolayer 1T-$MoTe_2$ (same as Figure 2h in the main text). b, c) Calculated band structures that take into account the band folding with the periodicity of (2√3×2√3)R30° for 1H, and 1T monolayer $MoTe_2$, respectively.

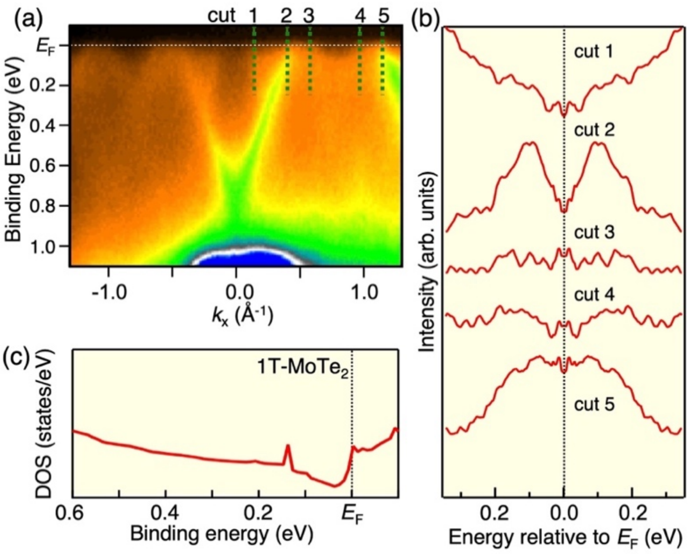

Figure S9. Energy-gap opening in monolayer 1T-$MoTe_2$. a) ARPES intensity at T = 40 K measured along the ΓM cut (k_x cut) of graphene BZ. b) Symmetrized EDCs obtained at five representative **k** points (cuts 1-5 in A). c) Calculated DOS of free-standing monolayer 1T-$MoTe_2$.

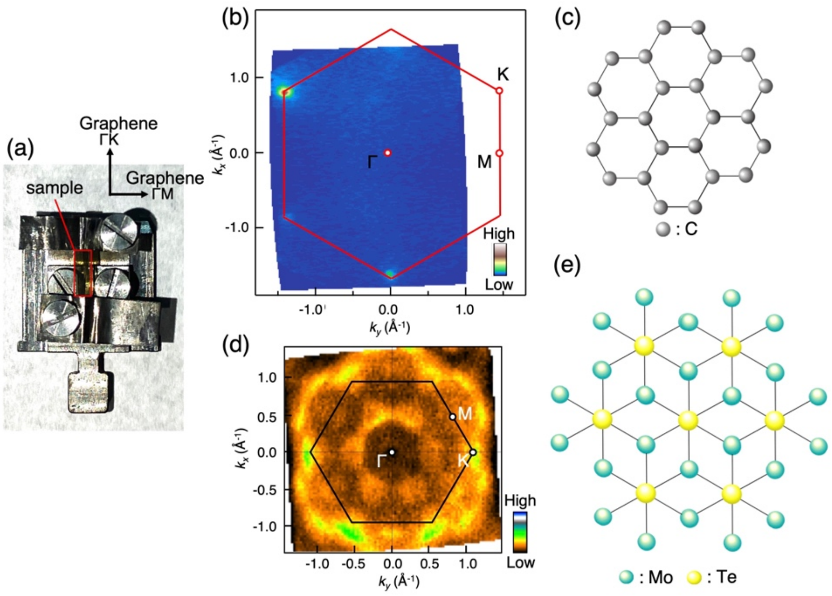

Figure S10. Relationship between sample geometry and Fermi-surface mapping. a) Photograph of sample holder for the epitaxial growth and ARPES measurement in which the SiC(0001) substrate is attached. Geometry of graphene (ΓK or ΓM) is indicated by arrows. b, c) Result of FS mapping for bilayer graphene on SiC(0001) and corresponding crystal geometry of graphene, respectively. d, e) Same as (b) and (c) but for monolayer 1T-$MoTe_2$.

	1H phase	1T phase	1T' phase
Atomic positions (bohr)	Mo (0.0000, 0.0000, 0.0000)<br>Te (1.1997, 2.399,1.79591)<br>Te (1.1997, 2.3993,-1.7959)	Mo (0.0000, 0.0000,0.0000)<br>Te (1.1729, 2.3459, 1.8589)<br>Te (2.3459, 1.1729, -1.8589)	Mo (0.0000, 3.4607, 0.0943)<br>Te (-2.4860, 3.4607, 1.4795)<br>Te (0.6553, 1.7304, 2.0737)<br>Mo (2.3113, 1.7304, -0.0943)<br>Te (1.6556, 3.4607, -2.0737)<br>Te (-1.5877, 1.7304, -1.4796)
Lattice constants (Å)	a = 3.5990<br>b = 3.5990	a = 3.5188<br>b = 3.5188	a = 6.3851<br>b = 3.4607

Table S1. Atomic arrangement and lattice constant of monolayer 1H, 1T, and 1T' $MoTe_2$ adopted in the present DFT calculations. These parameters were obtained after the structural optimization.

SI references

[1] S. Tang, C. Zhang, C. Jia, H. Ryu, C. Hwang, M. Hashimoto, D. Lu, Z. Liu, T. P. Devereaux, Z.-X. Shen, S.-K. Mo, *APL Mater*. **2018**, 6, 2.

[2] R. Castelino, T. T Pham, A. Felten, R. Sporken, *Nanotech.* **2019**, 31, 115702.

[3] T. T. Pham, R. Castelino, A. Felten, R. Sporken, *Appl. Surf. Sci.* **2020**, 523, 146428.

[4] D. Xiao, G.-B. Liu, W. Feng, X. Xu, W. Yao, *Phys. Rev. Lett.* **2012**, 108, 196802.

[5] T. Kawakami, K. Sugawara, T. Kato, T. Taguchi, S. Souma, T. Takahashi, T. Sato, *Phys. Rev. B* **2021**, 104, 045136.

[6] C. Zhang, W. Liu, F. Zhan, T. Zhang, L. Liu, M. Zhang, S. Xie, Z. Li, H. Sang, H. Ge, Y. Yan, R. Wang, Y. Wang, Q. Zhang, X. Tang, *Adv. Func. Mater.* **2021**, 31, 2103384.

[7] T. T. Pham, P. Vancsó, M. Szendrő, K. Palotás, R. Castelino, M. Bouatou, C. Chacon, L. Henrard, J. Lagoute, R. Sporken, *npj 2D Mater. Appl.* **2022**, 6, 48.

[8] X. Qian, J. Liu, L. Fu, J. Li, *Science* **2014**, 346, 6215.

[9] S. Barja, S. Wickenburg, Z.-F. Liu, Y. Zhang, H. Ryu, M. M. Ugeda, Z. Hussain, Z.-X. Shen, S.-K. Mo, E. Wong, M. B. Salmeron, F. Wang, M. F. Crommie, D. F. Ogletree, J. B. Neaton, A. Weber-Bargioni, *Nat. Phys.* **2016**, 12, 751-756.

[10] J. Zhang, Y. Xia, B. Wang, Y. Jin, H. Tian, W. k. Ho, H. Xu, C. Jin, M. Xie, *2D Mater.* **2021**, 8, 015006.
